# Influence maternal background has on children’s mental health

**DOI:** 10.1186/s12939-017-0559-1

**Published:** 2017-04-18

**Authors:** Elena Arroyo-Borrell, Gemma Renart, Carme Saurina, Marc Saez

**Affiliations:** 1 Research Group on Statistics, Econometrics and Health (GRECS), University of Girona, Carrer de la Universitat de Girona 10, Campus de Montilivi, 17003 Girona, Spain; 2 CIBER of Epidemiology and Public Health (CIBERESP), Madrid, Spain

**Keywords:** Maternal factors, Children, Mental health, SDQ, Spain, C1, C2, I1

## Abstract

**Background:**

In this paper, we aim to discern how a mother’s health and her socioeconomic determinants may influence her children’s mental health. In addition to this, we also evaluate the influence of other household characteristics and whether or not the economic downturn has heightened the effect a parent’s social gradient has on their children’s mental health.

**Methods:**

We use samples comprised of 4-14-year-old minors from the 2006 Spanish National Health Survey (SNHS), undertaken prior to the crisis, and the 2011 SNHS, carried out during the crisis. The participating children’s mental health is assessed using the Strengths and Difficulties Questionnaire (SDQ). Mixed models are used to evaluate the influence a mother’s health and her socioeconomic status may have on her children’s mental health. We also add interactions to observe the effect specific socioeconomic determinants may have had during the economic downturn.

**Results:**

The risk of a child suffering from mental health disorders increases when their mother has mental health problems. Socioeconomic determinants also play a role, as a low socioeconomic status (SES) increases the risk of a child exhibiting behavioural problems, being hyperactive or antisocial, whereas when a mother has attained a high level of education, this significantly reduces the probability of a child having mental health problems. ‘Homemaker’ is the activity status most positively related to children’s mental health. The findings show that the Spanish economic downturn has not significantly changed children’s mental health problems and the negative effects of low maternal SES are no greater than they were before the crisis. The main difference in 2011, with respect to 2006, is that the risk of children suffering from mental health problems is higher when their parents are (long or short-term) unemployed.

**Conclusions:**

In conclusion, both a mother’s health and her socioeconomic status, as well as other household characteristics, are found to be related to her children’s mental well-being. Although the crisis has not significantly changed mental health disorders in children or the social gradient of parents in general, at-risk children are the most negatively affected in the Spanish economic downturn.

## Background

According to the World Health Organization (WHO) 10–20% of children and adolescents around the world suffer from mental health problems, and their healthy development and productive lives in the future is of special concern [1, 2]. However, when only the most severe cases are considered, children and adolescents suffering from mental health problems is about 4–6% [3]. Half of all mental health problems begin during childhood [4]. In the US alone, child and adolescent mental health problems cost US $247 billion annually [5] and, more importantly, an improvement in the mental health of only one child can save US $140.000 over the lifetime of that child [6]. In Spain, one million children and adolescents suffered from mental health disorders in 2015 [7]. The main results from the Health Survey of Catalonia using the Strengths and Difficulties Questionnaire (SDQ) [8], show that the likelihood of reporting a mental health problem is about 3.8% in 4–15-year-olds, whereas in 2006 it was about 6.8%. However, over the last five years the number of children and adolescents attending a Children and Young People’s Mental Healthcare Centre (CSMIJ) with a severe mental health problem has increased by 53% [9].

As such, parental circumstances are crucial in children’s growth and development [10] and, since studies have argued that maternal factors are the strongest predictor of negative outcomes for children [11–14], it is essential to study the role a mother in particular plays in the mental health of her child. However, despite this need, very little literature has focused on how a mother’s health and/or health behaviour are associated with her children’s mental health [15, 16]. In the specific case of Spain, few studies have analysed the influence of maternal socioeconomic determinants (SES) on a child’s mental well-being [17].

In general, maternal health disorders are associated with poorer health consequences in the children [16]. Most of the literature initially discusses the effects maternal mental health during pregnancy or postpartum has on children [18, 19] and then, given the importance of the mother’s role as her child’s caregiver (e.g. providing food and protection), further attention is placed on how maternal mental health affects children’s mental health [20, 21]. Thus, a child whose mother suffers from depression will have higher rates of disruptive behaviours in preschool [22] and, more particularly, a higher ratio of emotional symptoms, problems with personal conduct and difficulties with their peers [23, 24]. Using data from longitudinal studies, Cents et al. [25] report that children whose mothers have higher trajectories of depressive symptoms have significantly more behavioural problems.

Furthermore, a family’s low SES is also considered to pose a risk to a child’s health. The literature agrees that families from low socioeconomic communities in particular are considered a risk factor for the mental health of the child [26, 27], and studies using Spanish data also reach the same conclusion [28, 29]. Moreover, maternal SES is of particular interest as mothers tend to be more efficient at taking care of their children [30]. As Feinstein et al. [30] claim, more highly educated mothers are able to be more effective in providing the social support required to cope with the effect a mother’s mental health disorders have on her children’s health. Kalff et al. [31] observe that low levels of parental education and occupation have an impact on children’s behavioural problems. In Spain, Sonego et al. [17] show a strong association between parental education and a child’s mental health, especially when maternal education is analysed. In line with Sonego’s results, children whose mothers have lower levels of education have been demonstrated as tending to have poorer mental health [28]. However, very little literature focuses how both maternal health and maternal socioeconomic status can affect specific mental health outcomes in children.

This paper aims to clarify how such conditions, (we are particularly interested in the mental health and the socioeconomic status of mothers ), are associated with the mental well-being of their children. In addition to this, we assess the influence other household characteristics may, or may not have, and whether or not the economic downturn has heightened the effect a parent’s social gradient has on their children’s mental health.

## Methods

### Study population

We use individual-level data from the face-to-face cross-sectional population-based Spanish National Health Survey (SNHS) for two periods: 2006 (prior to the economic downturn) and 2011 (in the middle of the economic crisis). Every five years the SNHS is conducted jointly between the Ministry of Health, Social Services and Equality (MSSSI) and the National Statistics Institute (INE) and is a stratified tri-stage sample [32]. The first stage is the census tract and the second unit is the main family residence. The survey consists of three questionnaires: one for households, another for adults and a third for minors (aged 0 to 15 in SNHS-2006 and aged 0 to 14 in SNHS-2011). One adult is selected from each household to fill out the Adult’s Questionnaire and, should there be any minors, one is (randomly) selected to fill out the Minor’s Questionnaire. The survey is independent and representative of each Spanish region (Autonomous Communities). SNHS-2006 was conducted from June 2006 to June 2007 and the sample included 9122 interviews concerning children and answered by either their mother, father or guardian. SNHS-2011 was carried out from July 2011 to June 2012, with 5495 children interviews.

### Instrument: Strengths and Difficulties Questionnaire (SDQ)

Goodman [33] developed the Strengths and Difficulties Questionnaire (SDQ) to satisfy the clinical need for a short and useful questionnaire that is well accepted by respondents [34]. The Questionnaire (SDQ) has allowed researchers to elucidate factors related to children’s mental health [33, 35] and it has been widely used and validated across different countries and cultures [34]. In Spain, in particular, the SDQ was validated by Rodríguez-Hernández et al. [36] and since 2006 it has been included in the Spanish National Health Survey (SNHS). Nevertheless, most of the research done to date has been focused on the Total Difficulties Score (TDS-SDQ) [28, 29, 37, 38] and few studies use the desegregated information from the five scales of mental health that the SDQ provides [39–41], which would provide more specific results to better address children’s mental health issues.

For the response variables, we used an SDQ questionnaire. SDQ is aimed at assessing the mental health of children, in our case based on the parents’ responses, and is widely used in research [40]. The questionnaire includes 25 statements about the child which are divided into five scales: four negative scales (emotional symptoms, behavioural problems, hyperactivity and problems with peers) and one positive scale (prosocial behaviour). Each scale has five questions, each of which is valued as 0, 1 or 2, for “not true”, “somewhat true” and “certainly true” (3-point Likert-type scale). Therefore, the sum of each scale is valued 0 to 10, from good to poor mental health (and for the positive scale it is from poor to good mental health). In order to analyse all five scales to assess the mental health problems of the child, first we categorise each scale as a binary variable (where 1 indicates “probable cases” and 0 “no cases”), in accordance with Goodman [33], Fleitlich et al. [34], the Ministry of Health, Social Services and Equality [40], and Children of Parents with a Mental Illness (COPMI) [42], albeit bearing in mind we are interested in the antisocial behaviour rather than the prosocial behaviour as it is defined in SDQ. More detailed information is provided in Table 1.

**Table 1:** Variables used in the analysis

Variable role	Definition and comments
Binary dependent variables	Emotional symptoms. The five questions are: “Often complains of headaches, stomach-ache or sickness”; “Many worries, often seems worried”; “Often unhappy, down-hearted or tearful”; “Nervous or clingy in new situations, easily loses confidence”, and “Many fears, easily scared”. Valued as one if the points is four or more.
	Behaviour problems. The five questions are: “Often has temper tantrums or hot tempers”; “Generally obedient, usually does what adults request”; “Often fights with other children or bullies them”; “Often lies or cheats”; and “Steals from home, school or elsewhere”. Valued as one if the punctuation is three or more.
	Hyperactivity. The five questions are: “Restless, overactive, cannot stay still for long”; “Constantly fidgeting or squirming”; “Easily distracted, concentration wanders”; “Thinks things out before acting”; and “Sees tasks through to the end, good attention span”. Takes the value of one if the punctuation is six or more.
	Peer problems. The five questions are: “Rather solitary, tends to play alone”; “Has at least one good friend”; “Generally liked by other children”; “Picked on or bullied by other children”; and “Gets on better with adults than with other children”. Takes the value one if the punctuation is three or more.
	Prosocial behaviour. The five questions are: “Considerate of other people's feelings”; “Shares readily with other children (treats, toys, pencils, etc.)”; “Helpful if someone is hurt, upset or feeling ill”; “Kind to younger children”; and “Often volunteers to help others (parents, teachers, other children)”. As we analyse the antisocial behaviour, the variable takes the value of one if the punctuation is below six.
Main independent variables	Maternal health variables: question on self-rated health (SRH) status in the last twelve months; depression, anxiety or other mental health disorders during the last twelve months; prescription for tranquilizers in the last two weeks; prescription for antidepressants during the last two weeks; Mental health index using the Goldberg Health Questionnaire (GHQ-12) [43]; smoking behaviour categorised as smoker (reference category), occasional smoker, ex-smoker and non-smoker; hours of sleep; physical activity in the workplace categorised as sitting (reference category), standing up, walking and carrying things, or doing heavy tasks; BMI (WHO, 2016) categorized as: low and normal weight (BMI ≤ 25 kg/m2) (reference category), overweight (25 kg/m2 < BMI ≤ 30 kg/m2), obesity (BMI > 30 kg/m2) [44]. Both size and weight were reported by the individual; self-perception weight categorized as much more than normal (reference category), more than normal, normal and less than normal.
	Maternal socioeconomic determinants: age groups (15–35 years (reference category), 36–45, 45–55, 56–65, 66–75); nationality categorised as Spanish (reference category), foreign and Spanish, and foreign; marital status categorised as single (reference category), married, widowed, separated and divorced; social class of the household reference person categorised as three groups: I-II class (reference category), III class and IV-V-VI class [40, 45]; educational level categorised into four groups: no studies (reference category), primary school, secondary school, and tertiary education; current activity categorised as employed (reference category), retired, studying, homemaker, short-term unemployed and long-term unemployed; whether the mother is the household reference person.
	Household characteristics: number of children living in the household categorised as less than three (reference category), three or more than three; number of adults in the household (single parent, between three or four, more than four).
Covariates of the child	Child health variables: question on SRH reported by the main respondent in the last twelve months; reported chronic conditions e.g. allergy, asthma, diabetes, tumour, epilepsy or other illness; restriction of activities in the previous two weeks; hours of sleep categorised as between eight and ten hours (reference category), less than eight, more than ten; physical activity habits categorised as no activity (reference category), occasionally, several times per month and several times per week; not having breakfast, eating fresh fruit more than three times per week; eating fast food more than three times per week; the mean of TV viewing hours watching or playing video games (separately) during the week and during the weekend (separately) categorised as none (reference category), less than three hours, between three and less than seven hours, seven hours or more; whether the child has been hospitalised in the previous year; number of GP visits during the last four weeks; number of specialist visits during the last four weeks; and BMI calculated dividing the weight in kilograms by the height in squared meters, and then using quintiles to better compare them [46].
	Sociodemographic characteristics: region of residence (17 autonomous communities); sex; age; and age groups (4–7 years, 8–10, 11–14).

As the questionnaire’s SDQ is aimed at 4 to 16-year-olds, we decide to take only those children aged between 4 and 14 (ages included in both periods of the SNHS-2006 and SNHS-2011). To collect information about the household and the adult, we take into account all three questionnaires. This, coupled with the fact that SDQ is more reliable with paternal responses, is why we only include those minors whose mother or father is the main respondent to the Adult’s Questionnaire. More specifically, as mother responses represent 72% of the sample, for the analysis we only consider characteristics related to the mother to obtain more statistical power. This resulted in 2761 minors in 2006 and 1339 in 2011.

### Main determinant: maternal and household characteristics

The main explanatory variables are the maternal and household characteristics that can be divided into maternal health variables, maternal socioeconomic determinants and household characteristics. For maternal health variables, we include self-rated health (SRH), diagnosed depression, prescription for tranquilizers or antidepressants, (divided separately), the Goldberg’s GHQ-12 mental health index, a counting variable ranging 0–12 [43], smoking behaviour, hours of sleep, physical activity in the workplace, Body Mass Index (BMI) [44] and self-perception weight (more information in Table 1).

Maternal socioeconomic determinants include age group, nationality, marital status, social class of the household reference person [40, 45], categorised as high social class (includes I-II class and it is the reference category), middle social class (includes III class category) or lower social class (includes IV, V and VI categories), level of education, current activity status, and whether the mother is the household reference person. Finally, household characteristics are the number of children and the number of adults living in the household (when it equals to 1 it is considered a single-parent family) (see more information in Table 1).

### Covariates

The covariates included in the analysis are related to child health outcomes and health behaviour variables e.g. self-rated health (SRH) as reported by the main respondent, reported chronic conditions, restriction of activities in the previous two weeks, hours of sleep, physical activity, breakfast habits, fresh fruit eating habits, fast food eating habits, mean hours of TV viewing per week or weekend, (separately), mean hours of playing video games per week or weekend, (separately), whether the child has been hospitalised in the previous year, number of GP visits, number of specialist visits and the quintiles for the Body Mass Index (BMI) [46]. We also include the sociodemographic variables of the child such as the region of residence (autonomous communities), sex, age and age groups [40]. Finally, we also include the year of the survey (2006, before the financial crisis, or 2011, during the economic downturn).

### Statistical analysis

To estimate the probability of the corresponding response variable, emotional symptoms, behavioural problems, hyperactivity, problems with peers and antisocial behaviour occurring, we estimated mixed logistic regressions,

$$ \begin{array}{l}{\mu}_i=\mathrm{Prob}\left({y}_i=1\left|{X}_i,\beta \right.\right)\\ {} log\left(\frac{\mu_{1 i}}{1-{\mu}_{1 i}}\right)={X}_i^{\hbox{'}}\beta \\ {} Var\left({y}_i\left|{X}_i,\beta \right.\right)=\phi {\mu}_i\left(1-{\mu}_i\right)\end{array} $$

where y denotes the variable response, X a matrix of explanatory variables (containing the intercept, and interaction terms between the period and some SES variables e.g. the social class of the household reference person, mother’s education and mother’s current activity), β the associated vector of unknown parameters, *ϕ* is a dispersion parameter, since the real variance of the response often differs from the theoretical, and the subindex i denotes the individual.

The logistic regressions are ‘mixed’, because we include random effects in all models in order to capture the individual heterogeneity. We assume they are identical and independent Gaussian random variables with constant variance, i.e. *υ*_*jt*_ ~ *N*(0, *σ*_*υ*_^2^).

Because of the relative complexity of our models, inferences are performed using a Bayesian framework. This approach is considered the most suitable in accounting for model uncertainty [47], particularly that associated with the existence of individual heterogeneity. Furthermore, within the Bayesian approach, it is easy to specify a hierarchical structure on the (observable) data and (unobservable) parameters, all considered random quantities. It is important to note this fact because it implies that even when the random effects and regressors were correlated, estimators are consistent [48]. Within the (pure) Bayesian framework, we follow the Integrated Nested Laplace Approximation (INLA) approach [49, 50].

We use penalising complexity (PC) priors. These priors are invariant to reparameterisations and have robustness properties [51].

All analyses are made with the free software R (version 3.2.3) [52], available through the INLA library [49, 53].

## Results

The descriptive statistics of the sample of children aged 4–14 for each of the five mental health disorders analysed show that the most common mental health problem is hyperactivity (33.3% of the total sample), followed by behavioural problems (31.1%), emotional symptoms (19.3%), problems with peers (16.8%) and, finally, antisocial behaviour (7.7%). In 2011, all percentages are lower than those of 2006.

The models are estimated using a separate analysis for each scale. Table 2 shows the results from the variables of interest (i.e. maternal health variables, socioeconomic determinants and household characteristics) of the models. The results show that the determinants of the five conditions are not completely identical. A mother’s depression negatively affects emotional symptoms (OR = 1.20, 95% IC = [0.92–1.57]) and behavioural problems (OR = 1.25, 95% IC = [0.99–1.59]) of the child, but reduces antisocial behaviour (OR = 0.63, 95% IC = [0.39–1.04]). The risk of the child suffering mental health disorders increases when the mental health index of the mother is higher (which indicates poorer mental health), again with the exception of antisocial behaviour. In terms of smoking behaviour, results show that occasionally smoking reduces the risk of hyperactivity, smoking in the past reduces behavioural problems, hyperactivity or emotional problems, and mothers who are non-smokers reduce the probability of children experiencing emotional symptoms, having behavioural problems, being hyperactive or having peer problems in all cases when compared to mothers who are regular smokers. Overweight or obese mothers (using the BMI) lead to a significant reduction in the risk of the child of having behavioural problems or being hyperactive when compared to under- and normal-weight mothers. In terms of socioeconomic status, foreign nationality increases the risk of having peer problems by 70% (OR = 1.70, 95% IC = [1.25–2.32]) but, on the other hand, reduces the risk of being hyperactive or having behavioural problems (in the latter case, being Spanish or having foreign nationality have the same significant association). Furthermore, the social class of the family shows that lower socioeconomic status significantly increases the risk of the child being hyperactive, having behavioural problems, or exhibiting antisocial behaviour (apart from class III compared to class I-II where the risk of the child suffering from peer problems is improved). On the other hand, children whose mothers have a high level of education, compared to children with uneducated mothers, are less at risk from suffering from hyperactivity, emotional difficulties, behavioural problems, or conflicts with peers. The risk of the child suffering from hyperactivity, behavioural problems or exhibiting negative emotional symptoms is significantly reduced when the mother is a homemaker (OR = 0.76, 95% IC = [0.61–0.95]; OR = 0.75, 95% IC = [0.60–0.94]; OR = 0.76, 95% IC = [0.58–0.99], respectively). Retired mothers also decrease the risk of suffering from peer and emotional problems, and long-term unemployed mothers reduce the risk of behavioural problems. Meanwhile, mothers who are studying increase the risk of hyperactivity (OR = 2.27, 95% IC = [0.84–6.15]). Finally, when the interactions between the period of the survey and some SES are added, the results show that in 2011 the risk of a child suffering from hyperactivity or emotional symptoms increases when the mother is a homemaker or retired (respectively) compared to 2006. In terms of the mothers being unemployed, in 2011 (compared to 2006) short-term unemployment increases the risk of having peer problems or antisocial behaviour. Long-term unemployment also increases the risk presenting behavioural problems as well as being hyperactive. Finally, in 2011, compared to 2006, the risk of suffering from an antisocial behaviour mental health disorder is reduced for low social classes and all mother levels studied. Furthermore, in 2011 the risk of hyperactivity and emotional problems in the classes IV, V, VI, is also reduced when compared to 2006.

**Table 2:** Mixed logistic estimations of children mental health

	Emotional symptoms	Behaviour problems	Hyperactivity	Peer problems	Antisocial behaviour
	OR (95% CI)	OR (95% CI)	OR (95% CI)	OR (95% CI)	OR (95% CI)
Health variables					
SRH (very good and good)					
Fair, bad and very bad SRH	0.92 (0.74–1.14)	0.94 (0.79–1.14)	1.10 (0.92–1.32)	1.07 (0.86–1.34)	0.83 (0.59–1.16)
Depression	1.20* (0.92–1.57)	1.25* (0.99–1.59)	1.14 (0.90–1.45)	1.18 (0.88–1.57)	0.63* (0.39–1.04)
Tranquilizer prescription(s)	1.18 (0.86–1.62)	0.95 (0.72–1.27)	0.88 (0.66–1.17)	1.04 (0.75–1.46)	1.25 (0.75–2.10)
Antidepressant prescription(s)	1.23 (0.83–1.82)	0.99 (0.69–1.42)	0.99 (0.69–1.42)	1.41* (0.93–2.12)	1.28 (0.63–2.60)
Mental health index	1.16** (1.12–1.20)	1.09** (1.06–1.13)	1.07** (1.04–1.11)	1.08** (1.04–1.12)	0.96 (0.90–1.03)
Smoker (yes)					
Yes, occasionally	1.10 (0.68–1.79)	0.90 (0.59–1.37)	0.56** (0.36–0.88)	1.25 (0.76–2.05)	0.83 (0.38–1.80)
In the past	0.83* (0.65–1.07)	0.80** (0.65–0.99)	0.85* (0.69–1.04)	0.96 (0.74–1.26)	1.02 (0.70–1.50)
No	0.79** (0.64–0.96)	0.65** (0.55–0.77)	0.75** (0.64–0.89)	0.83* (0.67–1.03)	1.11 (0.83–1.50)
Sleep (<=8 h)	1.11 (0.81–1.51)	1.08 (0.83–1.40)	0.99 (0.76–1.29)	1.04 (0.75–1.45)	0.51** (0.29–0.91)
Physical activity (sitting)					
Standing	1.33** (1.05–1.68)	0.98 (0.81–1.20)	1.13 (0.93–1.36)	0.99 (0.78–1.28)	1.13 (0.80–1.60)
Walking and carrying	1.26* (0.92–1.73)	0.95 (0.73–1.24)	1.16 (0.89–1.51)	1.41** (1.02–1.94)	1.05 (0.65–1.71)
Heavy tasks	1.51 (0.80–2.87)	1.79** (1.04–3.07)	0.99 (0.56–1.73)	1.68* (0.89–3.18)	0.49 (0.11–2.16)
BMI (under and normal weight)					
Overweight	1.05 (0.83–1.33)	0.81** (0.66–0.99)	0.73** (0.60–0.89)	0.95 (0.74–1.22)	1.08 (0.76–1.55)
Obese	0.83 (0.60–1.18)	0.71** (0.53–0.95)	0.75* (0.56–1.00)	1.11 (0.78–1.57)	1.41 (0.84–2.38)
Weight perception (much more than normal)					
More than normal	0.90 (0.64–1.27)	0.85 (0.63–1.14)	0.91 (0.68–1.21)	0.90 (0.63–1.29)	1.22 (0.69–2.15)
Normal	0.72* (0.50–1.04)	0.69** (0.50–0.95)	0.69** (0.50–0.95)	0.86 (0.58–1.26)	1.52* (0.83–2.79)
Less than normal	0.75 (0.44–1.27)	0.79 (0.50–1.25)	0.97 (0.62–1.52)	0.99 (0.57–1.73)	1.95* (0.84–4.50)
Socioeconomic determinants					
Age (15–35 years old)					
36–45 years old	0.91 (0.72–1.15)	1.01 (0.83–1.23)	0.76** (0.63–0.93)	0.89 (0.70–1.13)	1.15 (0.81–1.63)
46–55 years old	0.93 (0.67–1.29)	0.87 (0.66–1.15)	0.79* (0.60–1.03)	0.98 (0.70–1.38)	0.88 (0.53–1.46)
56–65 years old	1.31 (0.20–8.69)	0.85 (0.16–4.62)	0.30 (0.03–2.53)	2.46 (0.44–1.38)	1.12 (0.11–15.81)
66–75 years old	0.03 (2.4e-6–436)	0.02 (2.5e-6–183)	0.03 (2.4e-6–482)	0.16 (1.2e-6–2.1e4)	39.98 (0.01–118530)
> 75 years old	0.19 (9.9e-7–3.65e4)	0.11 (1.7e-6–7129)	0.17 (1.2e-6–2.3e4)	0.19 (1.0e-7–3.3e4)	0.92 (5.9e-9–1.4e08)
Nationality (Spanish)					
Foreign	0.84 (0.60–1.17)	0.82* (0.62–1.09)	0.81* (0.61–1.07)	1.70** (1.25–2.32)	0.80 (0.48–1.33)
Foreign and Spanish	1.00 (0.51–1.96)	0.57* (0.30–1.10)	0.81 (0.44–1.49)	1.01 (0.50–2.04)	1.20 (0.43–3.37)
Marital status (single)					
Married	0.81 (0.59–1.12)	0.83* (0.63–1.09)	0.79* (0.60–1.04)	0.69** (0.50–0.95)	0.98 (0.57–1.68)
Widowed	0.69 (0.33–1.43)	0.74 (0.39–1.41)	0.37** (0.18–0.75)	0.93 (0.45–1.91)	0.80 (0.16–3.94)
Separated	0.84 (0.52–1.33)	0.88 (0.59–1.32)	0.87 (0.58–1.31)	0.81 (0.50–1.31)	1.21 (0.54–2.70)
Divorced	0.68* (0.42–1.08)	0.95 (0.64–1.41)	1.02 (0.69–1.51)	0.82 (0.52–1.31)	2.21** (1.04–4.72)
Social class (Class I, II)					
Class III	1.02 (0.73–1.42)	1.09 (0.83–1.43)	1.24* (0.95–1.62)	0.75* (0.53–1.05)	1.78** (1.15–2.74)
Class IV, V, VI	1.24 (0.91–1.69)	1.29** (1.00–1.66)	1.58** (1.23–2.02)	1.01 (0.74–1.37)	1.73** (1.13–2.63)
Education (none)					
Primary	0.62* (0.38–1.02)	0.77 (0.49–1.20)	0.69* (0.44–1.07)	0.69* (0.42–1.14)	0.66 (0.33–1.31)
Secondary	0.67* (0.39–1.13)	0.62* (0.40–1.03)	0.67* (0.42–1.06)	0.69* (0.40–1.17)	0.87 (0.42–1.80)
Tertiary	0.59* (0.34–1.03)	0.53** (0.32–0.86)	0.58** (0.36–0.95)	0.75 (0.43–1.31)	1.02 (0.48–2.17)
Current activity (working)					
Retired	0.38* (0.13–1.12)	1.10 (0.50–2.44)	0.98 (0.42–2.24)	0.39* (0.12–1.27)	0.02 (1.4e-5–22.64)
Studying	1.17 (0.38–3.63)	0.73 (0.26–2.05)	2.27* (0.84–6.15)	0.70 (0.22–2.31)	0.02 (2.8e-6–161.3)
Homemaker	0.76** (0.58–0.99)	0.75** (0.60–0.94)	0.76** (0.61–0.95)	1.04 (0.79–1.36)	1.11 (0.78–1.57)
Unemployed short-term	0.95 (0.60–1.48)	0.88 (0.59–1.30)	1.27 (0.87–1.85)	0.94 (0.58–1.52)	0.99 (0.51–1.93)
Unemployed long-term	0.91 (0.54–1.53)	0.69* (0.44–1.09)	0.78 (0.51–1.20)	0.89 (0.52–1.52)	0.77 (0.36–1.65)
Person of reference	1.26* (0.99–1.60)	1.08 (0.88–1.33)	1.15* (0.94–1.41)	1.37** (1.07–1.76)	0.95 (0.66–1.36)
Household characteristics					
Number of minors in the household (<3)					
Three	0.83 (0.57–1.22)	1.16 (0.86–1.57)	0.98 (0.72–1.34)	0.94 (0.64–1.37)	1.64** (1.02–2.63)
More than three	1.21 (0.52–2.80)	1.58* (0.79–3.19)	1.51 (0.75–3.02)	1.00 (0.42–2.38)	0.60 (0.13–2.75)
Number of adults in the household					
Single parent	0.99 (0.69–1.42)	1.04 (0.76–1.42)	0.93 (0.68–1.27)	0.70* (0.48–1.01)	0.51** (0.27–0.98)
Between 3 and 4	0.99 (0.80–1.24)	1.00 (0.83–1.21)	1.13* (0.94–1.36)	1.11 (0.88–1.39)	1.42** (1.04–1.94)
More than 4	0.90 (0.44–1.84)	1.02 (0.57–1.83)	1.09 (0.61–1.93)	1.16 (0.60–2.24)	1.03 (0.40–2.67)
Interactions					
period*retired	4.94* (0.61–39.97)	0.99 (0.16–6.14)	1.20 (0.17–8.57)	0.03 (2.5e-6–258)	43.59 (0.01–1.3e5)
period*studying	0.07 (2.2e-6–2.4e3)	0.05 (2.4e-6–877)	1.28 (0.06–25.99)	0.23 (7.8e-7–6.6e4)	0.99 (3.2e-9–3e8)
period*homemaker	1.36 (0.85–2.12)	1.25 (0.84–1.86)	2.03** (1.37–3.00)	0.90 (0.53–1.52)	1.26 (0.55–2.89)
period*unempl short-term	1.11 (0.56–2.18)	0.95 (0.53–1.73)	0.84 (0.47–1.49)	2.00** (1.00–4.00)	2.23* (0.72–6.91)
period*unempl long-term	0.91 (0.44–1.88)	1.74* (0.95–3.19)	1.67* (0.92–3.01)	1.64* (0.78–3.44)	2.27* (0.69–7.53)
period*Class III	0.89 (0.49–1.62)	0.83 (0.50–1.38)	0.82 (0.50–1.34)	1.26 (0.65–2.43)	0.43* (0.15–1.22)
period*Class IV,V,VI	0.70* (0.41–1.20)	0.79 (0.50–1.24)	0.68* (0.44–1.06)	0.87 (0.48–1.56)	0.31** (0.12–0.79)
period*primary	1.23 (0.38–3.97)	0.85 (0.30–2.35)	1.51 (0.51–4.46)	0.73 (0.22–2.36)	0.20** (0.04–0.91)
period*secondary	1.15 (0.35–3.84)	0.81 (0.29–2.31)	1.28 (0.43–3.87)	0.76 (0.23–2.56)	0.09** (0.02–0.48)
period*tertiary	0.98 (0.28–3.40)	0.62 (0.21–1.83)	1.48 (0.48–4.56)	0.44 (0.13–1.57)	0.07** (0.01–0.40)

*p < 0.1, **p < 0.05; The model is adjusted by all the covariates of the child and the explanatory variables

The results from the mixed logistic estimations of covariates concerning the children’s mental health are available upon request.

## Discussion

This paper’s main contribution to the literature is identifying that SES and maternal health (specifically mental health) are mutually associated with children’s health. Previous research determining that the mental health of the child worsens with low SES or paternal mental health, has treated these two areas separately, but the mutual association (i.e. considering both elements for the mother) when clarifying the main factors related to a child’s mental health is important because the subsequent consequences of that child’s mental well-being may be far-reaching and even have an impact on their adulthood. Furthermore, the need for more research into this area is easily justified by the economic costs associated with mental health problems in adolescents and children [54]. Moreover, we have been able to discern differences in the effects maternal SES has had on child mental health during the financial crisis and, although children’s mental health in general may not have deteriorated, there needs to be a clear focus placed on those children who are most at-risk and vulnerable.

As Hardie and Landale [16] also conclude, we find that socioeconomic and maternal health factors are associated with children’s health outcomes. More specifically, maternal mental health, such as depression, is associated with higher risks of emotional and behavioural problems, and the mental health index is also associated with hyperactivity and peer problems [22–25]. In general, from our results overall in the not-smoking and smoking categories are compared, we can draw the conclusion that mothers who smoke occasionally, used to smoke or do not smoke reduce the likelihood of a child suffering from mental health disorders. A recent study carried out in Spain [55] also supports this finding. However, we have not found any evidence supporting that being an obese or overweight mother, (as opposed to those classified as normal weight), would play any kind of protective role in a child’s metal well-being. In fact, most of the literature agrees with the negative effects that being obese or overweight during pregnancy has on children’s mental health. When observing the impact a mother’s nationality has on her children’s mental health in Spain, being a foreign national plays a more protective role compared to being a Spanish national. Here, although the literature finds mixed results, in our case it would seem that the higher levels of psychological well-being are greater than the poorer psychological outcomes that these children may experience.

Alongside this, SES is associated with children’s mental health and this relationship between the social class of the breadwinner and the general mental health of the child has been demonstrated using the SDQ-TDS [28, 40]; albeit not for specific mental health problems. As our results show, the lowest, when compared to the highest, socioeconomic group indicates that the risk of having behavioural problems or hyperactivity increase for children from lower socioeconomic backgrounds. In terms of a mother’s level of education, we find that a low level of education is positively strongly associated with all mental health disorders of a child [17, 28, 55], albeit with the exception of antisocial behaviour. Thus, our results are also consistent with Barriuso-Lapresa et al. [28] who also draw attention to the importance of the social gradient in terms of maternal education and her family’s socioeconomic circumstances. In terms of a mother’s current status, the role maternal employment has on a child’s mental health has scarcely been studied. One of the few studies available is that of Mukherjee and Fink [56], and our results are in line with them because we also conclude that children whose mothers spend more time at home are less likely to suffer from mental health problems. As we have observed, mothers who are retired or homemakers decrease the risk of the child suffering from some mental health disorders; as do long-term unemployed mothers who slightly decrease the risk of suffering from behavioural problems.

As Golberstein et al. [57] point out, the literature about the effects economic conditions have on children’s mental health is scarce. However, concern about economic conditions and children’s health is well established [58, 59]. In this paper, we conclude that during the Spanish economic downturn, the negative effects of low maternal SES do not appear to be greater when compared to before the crisis. This would mean that the financial crisis has not widened the differences between social classes, maternal levels of education or maternal current activity status. In fact, the main difference in 2011, with respect to 2006, is that the risk of children suffering from mental health problems is higher if their mothers are unemployed (long or short-term); just as Rajmil et al. [38] also concluded for the unemployment of all family members. So, while the gradient of social class or maternal level of education has not widened, it has persisted [37].

This study faces some limitations that must be taken into consideration. First of all, it is worth mentioning that although SDQ highly correlates with outcomes such as mental health services and special education services [60], and it increases the ability to detect any psychiatric disorders a child may be suffering from as well as improving access to effective treatments [61], the questionnaire has some limitations that were acknowledged by its authors [33]. In addition, as the MSSSI report [40] also clarifies, the desegregated mental health problems and the categorization of the mental health disorders slightly reduces the performance of the SDQ tool. That said, our results are consistent with other similar findings. Secondly, as Goodman et al. [62] contemplate, the SDQ works better when the responses are from caregivers and teachers. This is the reason for only taking into account parents’ responses and taking out any other relationship between the adult and the child. However, in doing so the sample size has been considerably reduced and suffers from the disadvantages that causes. Thirdly, although some research is concerned about the influence of parents’ mental health disorders on the parent-reported mental health of the child, it has been proved that there is no relationship [58, 62, 63]. Besides, the correlations considered between SES and maternal health are controlled by the range of outcomes we introduced into the analysis. With the antisocial behaviour outcome, it is important to be prudent with the results as the statistical power is slightly reduced because less than 10% of the total sample suffers from antisocial behaviours. Finally, it is important to mention that the cross-sectional design of the SNHS has its limitations when assessing relationships and does not allow us to conclude direct relationships between maternal factors and children’s mental health.

## Conclusions

Both maternal health and SES, (along with other household characteristics) are found to be related to children’s mental health. Consequently, greater attention should be paid to those children whose mothers have poor mental health and low SES, given the long-term consequences that poor mental health in childhood can subsequently have in adulthood. In conclusion, although in general the financial crisis has not significantly changed the mental health disorders of children or the social gradient of parents, the most negatively affected during the Spanish economic downturn are those children who are most vulnerable because of their family situation. These findings suggest the need to formulate better public health policies, interventions and programs addressing this specific subgroup to protect and prevent them from the negative impacts of poor economic and health conditions.

## Abbreviations

BMI: Body Mass Index
COPMI: Children of Parents with a Mental Illness
CSMIJ: Children and Young People’s Mental Health Care Centre
GHQ-12: General Health Questionnaire
INE: National Statistics Institute
INLA: Nested Laplace Approximation
MSSSI: Ministry of Health, Social Services and Equality
OR: Odds ratio
PC priors: Penalising Complexity priors
SDQ: Strengths and Difficulties Questionnaire
SES: Socioeconomic status
SNHS: Spanish National Health Survey
SRH: Self-rated health
TDS-SDQ: Total Difficulties Score of the Strengths and Difficulties Questionnaire
WHO: World Health Organization
